# Effectiveness of Hands-Only Cardiopulmonary Resuscitation Teaching on Lay Bystander Attitudes Toward Future Resuscitation

**DOI:** 10.51894/001c.8749

**Published:** 2019-07-01

**Authors:** Jenna R. Nofzinger, Edward Kakish, Nael O. Bahhur, Joseph Ryno, Mahesh Pillai, Jessica DeBelly, Corion Jones

**Affiliations:** 1 Emergency Medicine Barnes-Jewish Hospital, St. Louis Missouri; 2 Emergency Medicine University of Toledo Health Science Campus (OH); 3 Family Medicine; 4 Human Research Protection Program University of Toledo Health Science Campus (OH); 5 Emergency Medicine The University of Toledo College of Medicine

**Keywords:** hands-only cardiopulmonary resuscitation, bystander cpr, cardiopulmonary resuscitation

## Abstract

**CONTEXT:**

It is estimated that there are approximately 1,000 sudden cardiac events occurring daily outside of the hospital setting in the US.

**OBJECTIVES:**

The objectives of this pilot project were to determine lay participants’ ability to remember the steps of hands-only cardiopulmonary resuscitation (HOCPR) following a 30-minute instructional session regarding proper technique and their willingness to perform bystander CPR (BCPR) on victims of sudden cardiac arrest outside in the community.

**SAMPLE AND SETTING:**

A nine-item survey questionnaire that was created by the authors was first administered to a sample of 75 adults who had volunteered at their institutions’ emergency department. Inclusion criteria included all adults over the age of eighteen years.

**OUTCOME MEASURES:**

To gauge whether basic HOCPR training improved bystander preparedness and willingness to provide assistance to a victim of sudden cardiac event.

**METHODS:**

After IRB approval, participants were shown a one-minute video by the American Heart Association (AHA) and provided a 30-minute demonstration of key HOCPR skills on a mannequin. A post-instruction nine-item survey was sent by mail/email or administered by phone at one month after training to assess participants’ retention of HOCPR knowledge and attitude.

**RESULTS:**

The initial survey responses showed 75 (100%) were able to recall the basic steps of HOCPR, with 59 (79%) “very likely” to help provide BCPR. Not needing to provide breaths made 57 (76%) of lay participants more willing to assist a person in need. A subgroup of 31 (41%) of the initial 75 participants were lost to follow-up. Out of the 44 (59%) who completed the one-month survey, 44 (100%) of participants remembered the primary HOCPR steps and technique. A subgroup of 32 (73%) one-month respondents indicated that they were more likely to assist victims if rescue breathing was not required, and 11 (25%) had reportedly tried to teach family and friends about HOCPR.

**CONCLUSIONS:**

These results indicate that of those involved in the survey, the majority could recall the correct steps and be willing to provide HOCPR. These results could help in shaping community outreach and training programs designed to improve the rate and quality of response to victims of sudden cardiac arrest.

## INTRODUCTION

Of the over 300,000 annual sudden cardiac arrest events occurring outside of US hospitals, only approximately one in four victims receive bystander cardiopulmonary resuscitation (BCPR).^1^ The studies to date have demonstrated that the use of hands-only cardiopulmonary resuscitation (HOCPR) after a cardiac event can double or triple a victim’s chances of survival when compared to when no cardiopulmonary resuscitation (CPR) has been performed.^2^ An observational 2007 study published compared patient outcomes after out of hospital cardiac arrest with bystander use of traditional CPR, HOCPR and no CPR at all. This study group found that either method to provide BCPR was associated with more favorable neurological outcomes than no action at all.^3^ The results of this study led the authors to question findings by other studies that have examined the attitudes bystanders have for or against providing BCPR.

A previous perceived barrier to administering BCPR has been fear of disease transmission secondary to the mouth-to-mouth ventilation. However, the results of a 2006 study showed that fear of causing further harm and a sense of panic also prevented many bystanders from performing CPR.^4^ However, fears of disease transmission was a much less significant concern of sample bystanders.^4^ This indicated to the authors that correction of misinformation regarding CPR and provision of simplified BCPR instructions could be incorporated when teaching BCPR to willing community members.

Three additional studies have demonstrated that HOCPR training does prospectively increase a bystander’s willingness to aid a victim of sudden cardiac arrest in community settings.^5–7^ In 2008, the American Heart Association (AHA) Emergency Cardiovascular Care Committee first issued an advisory advocating bystanders to use at least HOCPR on people suddenly collapsing.^5^ The current study reported in this paper was conducted to see if the results in our community would resemble the results in these aforementioned studies.

The purpose of this pilot study was two-fold. The first aim of the study to determine the ability of a sample of adult participants to remember the steps of HOCPR after being shown proper technique. The second aim was to measure the effect of teaching proper HOCPR technique on a layperson’s willingness to perform BCPR on a possible victim of sudden cardiac arrest. This was achieved by assessing sample participants’ knowledge, attitudes and willingness of participants to provide HOCPR, by administering surveys immediately (initial) and one month post-instruction HOCPR training. The authors’ overall null hypothesis was that they would measure no initial-to-one-month differences in participants’ survey scores.

## METHODS

Before patient recruitment or data collection, this study had been approved by the University of Toledo institutional review board. Eligible participants for the study included any adult over the age of eighteen. Willing participants were recruited from the relatives and friends of non-critical patients at the University of Toledo Medical Center Emergency Department (ED). If a lay person agreed to study participation, they were asked to sign an informed consent form. This informed consent form also included permission to contact the participants by mail, email or phone number to complete the one-month follow-up survey. A separate room in the ED had been set up for study recruitment.

After consent, a one-minute video clip from the AHA discussing the importance of HOCPR after a cardiac event to improve victim survival was shown followed by a 30-minute brief demonstration on a mannequin under provider supervision.^8^ This demonstration included the following: *Call 911, Push Hard, Push Fast* in center of the chest.

After mannequin practice, participants were then asked to complete the initial nine-item survey. Multiple study sessions needed to be scheduled to facilitate attendance by each of 75 study participants. The nine-item one-month survey was slightly different than the initial survey (copies of both surveys can be found at the end of the paper).

## RESULTS

Each of the 75 (46 females, 29 males) lay participants viewed the AHA training video and participated in the HOCPR demonstration and practice session. A total of 31 (41%) of the original 75 participants were lost to follow-up and 44 (59%) completed the one-month post-training survey. Socio-demographic information concerning the sample participants was obtained, but not analyzed for subgroup differences due to the size of the total study sample.

The initial survey conducted immediately following the video and practice session demonstrated that 100% of the participants (n = 75) were able to recall the basic steps of HOCPR. Of those 75 participants, 59 (79%) indicated that were very likely to help somebody in the event of a cardiac episode in the community. According to survey responses, the idea of not having to give breaths to the victim during BCPR made 57 (76%) participants more willing to assist a person in cardiac arrest.

One month after the initial training, the follow-up survey responses indicated that 44 (100%) of responding participants still remembered the various steps and compression techniques of HOCPR. Similar to the initial survey, 32 (73%) were more likely to assist a cardiac arrest victim in the community when no rescue breathing was involved. After learning the initial HOCPR content, 11 (25%) participants reported that they had tried to teach their family and friends about HOCPR.

## DISCUSSION

For this pilot project, we developed a straightforward training method for teaching participants the principles of HOCPR. Our instructional method was adapted from AHA guidelines for HOCPR^5^ and similar to that used in other studies.^2,9,10^ By conducting this study, we were able to initially demonstrate that HOCPR skills can be feasibly taught to a typical sample of lay community-dwelling adults that can be readily recalled by participants. Our results also indicate that by teaching HOCPR, and discussing the possibility of cardiac events, we were able to improve community bystander attitudes towards responding to any future cardiac arrest victims.

Our results are similar to results from a 2013 study that administered a prospective anonymous survey studying adult patients and visitors of an academic suburban ED indicating that only 23% of participants had prior knowledge of HOCPR.^11^ This group reported that after being educated on the utility of HOCPR techniques, most participants felt that they would be willing to help a victim of sudden cardiac arrest using BCPR.^11^ This notion that a simplified less invasive forms of bystander intervention has been shown to improves community members’ willingness to assist victims in an actual cardiac arrest has been replicated in a growing number of other studies.^6,9,12–14^

There remains a proportion of adults in the community who choose not to participate in BCPR due to fear of causing more harm.^1,4^ Although breaking a victim’s rib bones is a possibility, lay bystanders should be instructed that they cannot do more damage by beginning chest compressions in a person believed to be having a cardiac arrest.^1^ Our study found that learning HOCPR made many of our participants more likely to assist somebody in cardiac arrest. This finding could be explained by participants’ increased awareness of the need for BCPR in responders, their not needing to administer rescue breathing and a newly acquired confidence in their own ability to help a prospective cardiac arrest victim.

Similar results were found in a 2010 study which evaluated lay participants’ technique and willingness to perform BCPR after traditional instructor led training, video-based training, or no training at all.^7^ This earlier study showed a significant positive change in trainees’ willingness to perform bystander CPR. These results support the growing belief that increased awareness and education, in any form, can have a positive impact in the amount of bystander response to sudden cardiac arrests that occur in the community.^7^

Although our study did not evaluate the longer-term retention of participants’ skills for performing HOCPR, other research groups have investigated skill retention. For example, a 2014 study demonstrated skill retention was superior for the group taught compression only CPR versus the group taught traditional CPR.^10^ Their results showed that the number of appropriate chest compressions and the time without chest compressions was superior in the compression-only CPR group compared to the conventional CPR group.^10^ This type of skills training coupled with changes in community participants willingness should impact how members of the medical field approach lay first responder education.

An ongoing dilemma for first response educators arises between the desire to educate and increase the number of performers of BCPR, and to increase survival rates for cardiac arrest victims in the community. A 2011 study examined the relative outcomes and effectiveness of traditional CPR and HOCPR.^15^ This group analyzed over 40,000 cases of bystander witnessed cardiac arrest, and studied sub-groups of cardiac origin, non-cardiac origin, age, and time to CPR initiation. Most victims receiving traditional CPR proved to have better one-month survival and more favorable neurological outcomes than those who had received HOCPR.^15^ For both forms of CPR, a 10-minute or greater delay reduced neurologically favorable one-month survival in victims.^15^ This finding certainly suggests that if delivering traditional CPR is a barrier to responding, HOCPR is preferred over no CPR at all.

### Study Limitations

Although we re-evaluated participants’ retained HOCPR knowledge one month after training, we were unable to assess the participants’ ability to conduct safe and efficient HOCPR. A further limitation was that our results did not include assessment of longer-term retention of HOCPR. Our community-based convenience sample was quite small, and some sample participants were lost in follow-up. Our results may have been skewed by “self-selection” biases since those participants retained in the study may have neem more interested or had personal experiences with life threatening situations that caused them to be more interested in HOCPR training opportunities.

The study surveys were comprised of nine multiple-choice questions that the authors had created to test participant’s knowledge of HOCPR skills and techniques. Had a different style of evaluation been used (e.g., short answer or fill-in-the blank form), our results may have been different. During this project, we had not we taken into account sample participants’ earlier training use of HOCPR in emergent situations. Due to our study design, we were unable to draw conclusions about lay persons’ ease of learning HOCPR versus traditional CPR.

## CONCLUSION

Overall, our HOCPR training methods were simple and could be easily applied in other community and institutional training programs. Our results demonstrate that the provision of basic HOCPR training can improve bystander preparedness and willingness to provide assistance to future sudden cardiac event victims. Additionally, our follow-up surveys after the training session also indicate that participants could subsequently recall skills and techniques of HOCPR.

Larger-scale studies are needed to determine how BCPR training could be tailored for different lay learner groups to increase rates of bystander response through HOCPR for diverse cardiac arrest victim outcomes. Ideally, these study results can be used to design further community outreach HOCPR training programs to improve rates of bystander response to sudden cardiac events outside of the hospital setting.

### Conflict of Interest

The authors declare no conflict of interest.

## Supplementary Material

One month survey p. 1

One month survey p. 2

Initial survey p. 1

Initial survey p. 2
