# Supplementary figures and images for: Effectiveness of Hands-Only Cardiopulmonary Resuscitation Teaching on Lay Bystander Attitudes Toward Future Resuscitation
Source: Spartan Med Res J. 2019 Jul 1;4(1):8749. doi: 10.51894/001c.8749 (PMC7746055; doi:10.51894/001c.8749)

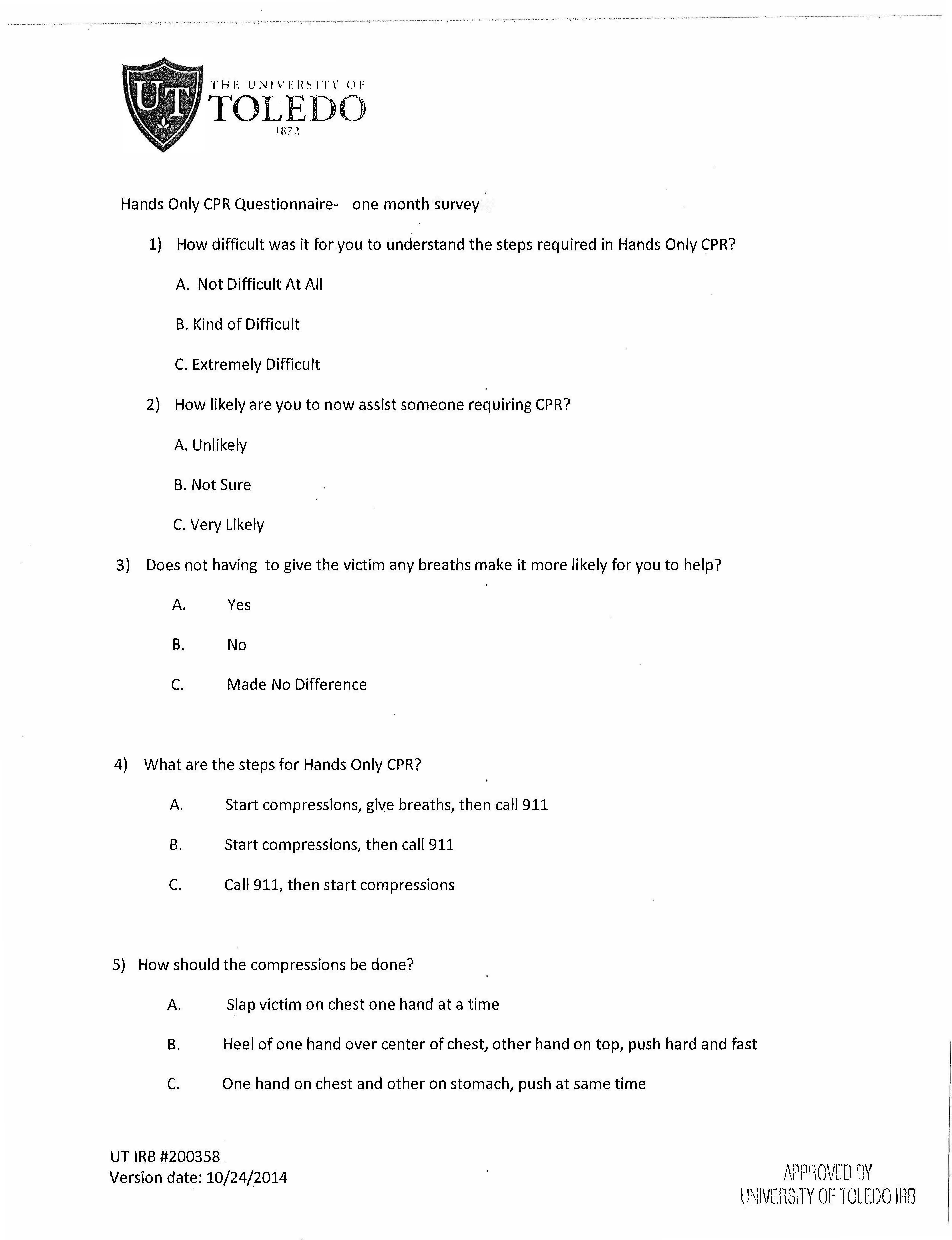

Supplement: One month survey p. [file smrj_2019_4_1_8749_21725.jpeg]

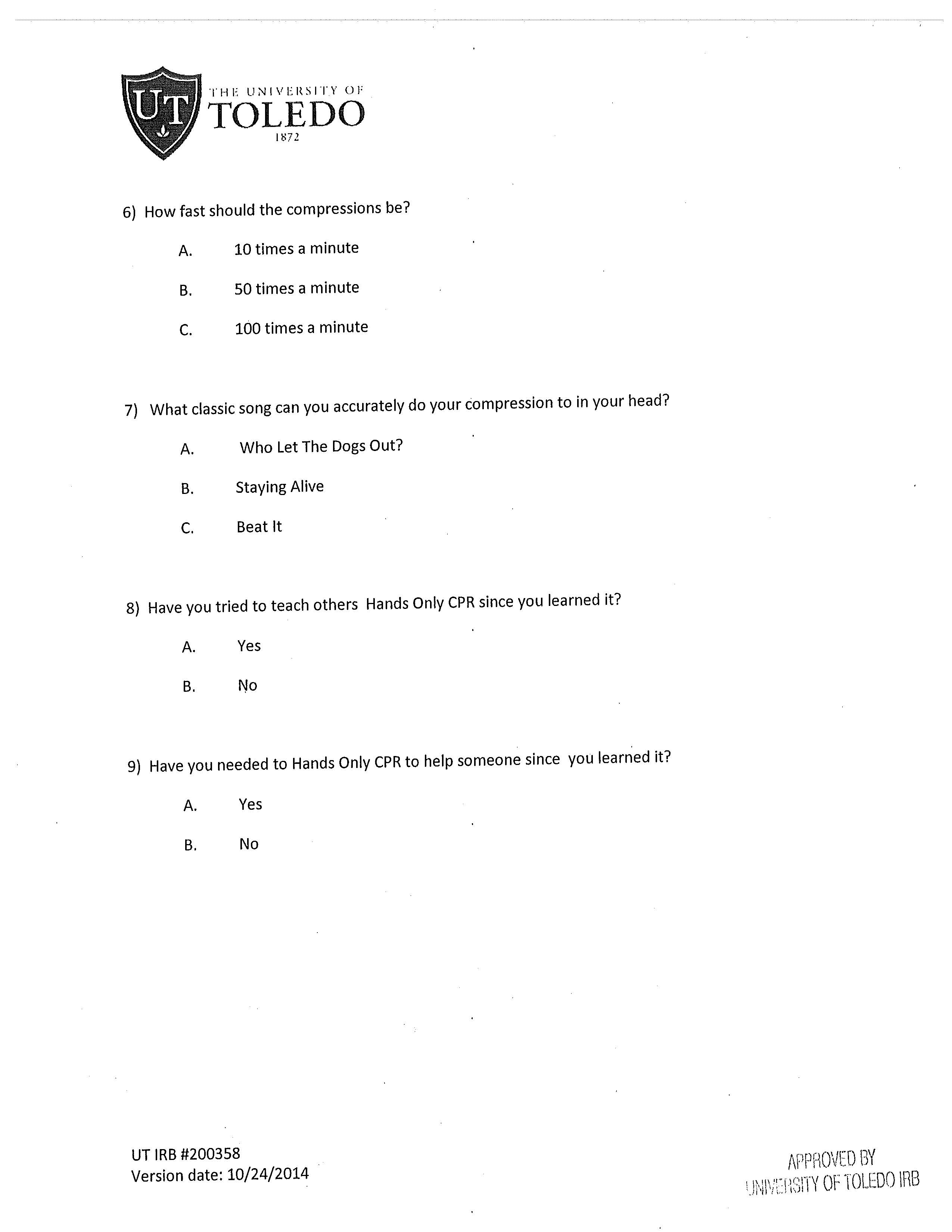

Supplement: One month survey p. [file smrj_2019_4_1_8749_21726.jpg]

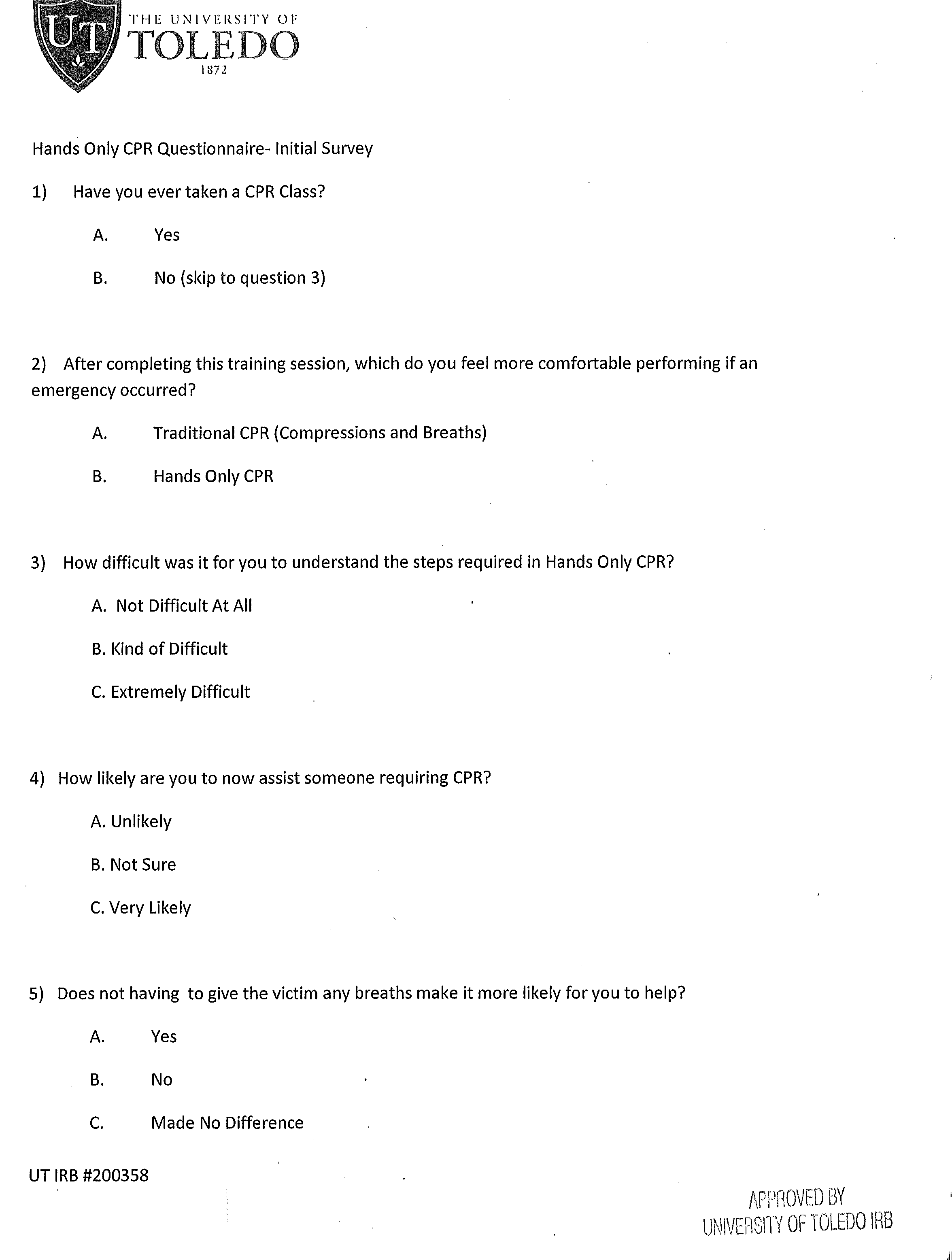

Supplement: Initial survey p. [file smrj_2019_4_1_8749_21736.png]

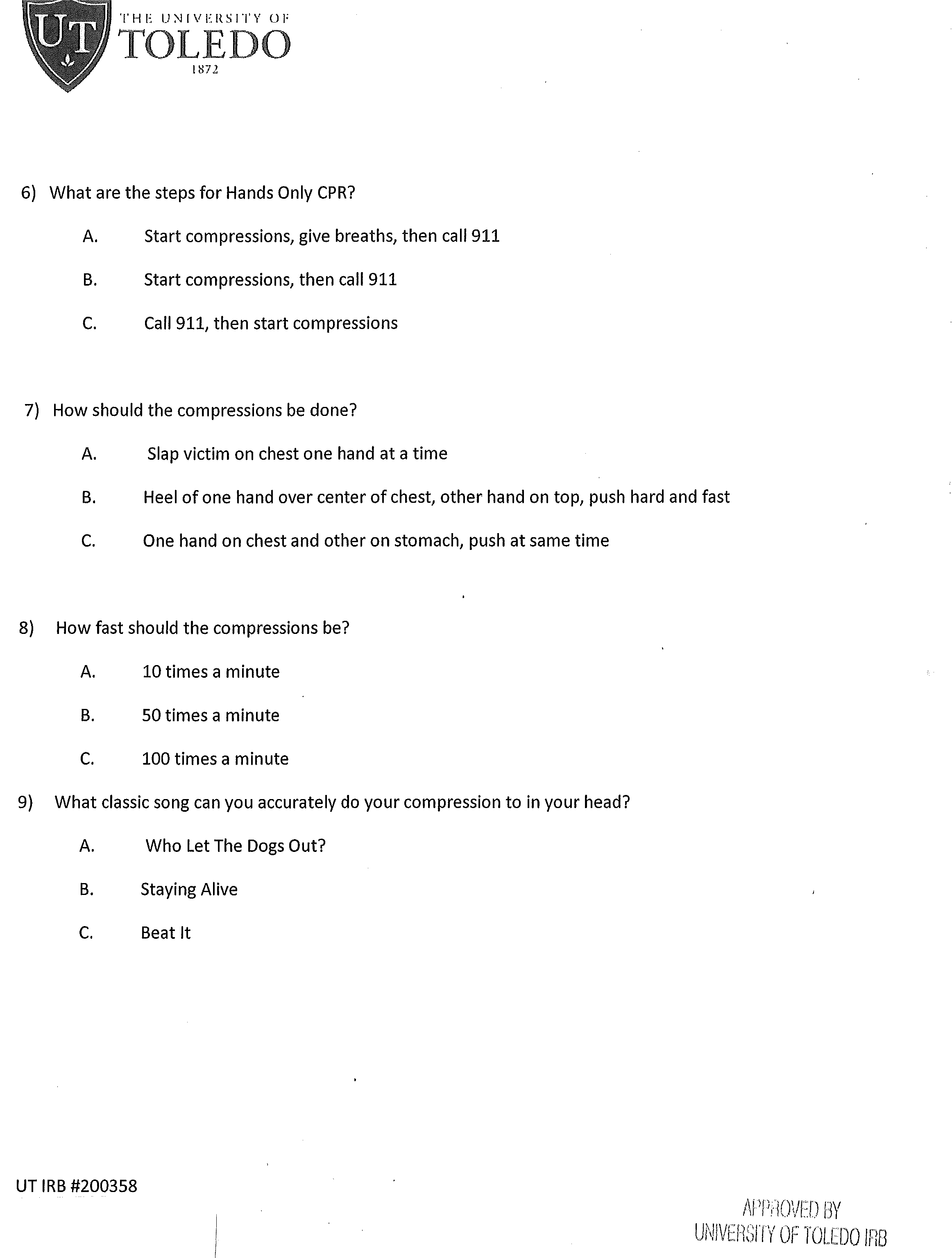

Supplement: Initial survey p. [file smrj_2019_4_1_8749_21737.png]
